# *Erodium stephanianum*-Derived Polyphenols Prevented the Progression of Collagen-Induced Arthritis in Mice and Its Associated Metabolic Alterations

**DOI:** 10.3390/nu18162683

**Published:** 2026-08-17

**Authors:** Aohua Kong, Fan Wang, Zongzhe Li, Qunqun Guo, Ke Xiong, Ronggui Li

**Affiliations:** 1 College of Life Sciences, Qingdao University, Qingdao 266075, China; kongaohua@qdu.edu.cn (A.K.); wangfan2@qdu.edu.cn (F.W.); gqunqun@qdu.edu.cn (Q.G.); 2 Institute of Nutrition and Health, School of Public Health, Qingdao University, Qingdao 266023, China; lizongzhe@qdu.edu.cn

**Keywords:** *E. stephanianum*, ellagic acid, collagen-induced arthritis, metabolomics

## Abstract

**Background:** This study aimed to investigate the effects of *E. stephanianum*-derived polyphenols on preventing the progression of collagen-induced arthritis (CIA) in mice and its associated metabolic alterations. **Methods:** The *E. stephanianum* plant was extracted, fractionated, and screened in vitro for anti-inflammatory, antibacterial, and antioxidant potential. Fraction 5 (identified as ellagic acid by nuclear magnetic resonance) exhibited the most potent activity and was selected for in vivo study. In the in vivo study, male DBA/1JGpt mice were randomized into five groups: control (CON), CIA (Model), low-dose extract (ES-L), high-dose extract (ES-H), and ellagic acid group. ES-L, ES-H, and ellagic acid were administered by daily oral gavage at 375, 750, and 250 mg/kg body weight, respectively, for 8 weeks. Arthritis severity was evaluated based on paw thickness, clinical score, ankle joint histopathology, and serum proinflammatory cytokine levels. To reveal the associated metabolic alterations, serum metabolites were characterized by ultra-performance liquid chromatography-quadrupole time-of-flight mass spectrometry. **Results:** Both the whole extract and ellagic acid revealed pronounced anti-inflammatory, antibacterial, and antioxidant properties. In vivo treatment with ES-L, ES-H, or ellagic acid similarly and significantly ameliorated CIA severity, showing smaller paw swelling, reduced clinical arthritis scores, milder joint histopathological injury, and lower serum tumor necrosis factor-α and interleukin-6 concentrations than the model group. Untargeted metabolomics identified 20 differential metabolites, mainly including triacylglycerols (containing *n*-3 fatty acids), pro-inflammatory phospholipids, and spermic acid 1. Moreover, the treatments significantly altered amino acid and purine metabolism. **Conclusions:** *E. stephanianum* derived polyphenols, like ellagic acid, significantly prevented the progress of CIA in mice. These effects were associated with the alteration of metabolic profiles.

## 1. Introduction

Rheumatoid arthritis (RA) affects approximately 0.5–1.0% of people worldwide, which is a chronic systemic autoimmune disease involving persistent synovitis, progressive cartilage and bone destruction, and substantial functional impairment [1,2]. Preventing RA has attracted increased research interest due to increased understanding of RA pathology and the ability to identify high-risk populations [3,4]. In recent years, phytochemicals have attracted increasing attention as potential adjunctive strategies for alleviating RA because of their actions on multiple targets and relatively favorable safety profiles [5].

In China, *Erodium stephanianum* (ES) has traditionally been used to alleviate rheumatic pain and inflammation [6]. Despite its traditional empirical application, direct evidence supporting the efficacy and mechanism of ES in RA remains scarce. This plant contains abundant phenolic acids, such as ellagic acid, gallic acid, and protocatechuic acid; flavonoids, including quercetin and kaempferol; and tannins, including corilagin and geraniin [7]. Among these constituents, corilagin and geraniin have been reported to regulate RA-related pathological processes, including oxidative stress, macrophage pyroptosis, NOD-like receptor family pyrin domain-containing 3 inflammasome activation, and nuclear factor-κB (NF-κB) and mitogen-activated protein kinase (MAPK) signaling [8,9,10]. However, it is not clear which component plays essential roles in attenuating RA.

RA is characterized not only by inflammation but also by extensive metabolic dysregulation, including alterations in energy metabolism, amino acid profiles, and lipid mediators, all of which are closely associated with disease onset and severity [11]. Because phytochemicals can act on multiple nodes within metabolic networks, they may exert preventive effects in RA through the restoration of metabolic homeostasis [12]. Untargeted metabolomics offers a valuable approach to investigate the mechanisms of phytochemicals in RA and to identify key metabolic targets.

In this study, ES was extracted and fractionated into six fractions (F1–F6), which were subjected to preliminary screening through in vitro antibacterial, antioxidant, and anti-inflammatory assays. We identified F5 as the most active fraction and characterized it as ellagic acid using nuclear magnetic resonance (NMR). Subsequently, ES and ellagic acid interventions were conducted in a well-established collagen-induced arthritis (CIA) mouse model, and their effects on preventing the progression of CIA were evaluated based on arthritic phenotypes, joint histopathological changes, and pro-inflammatory cytokine levels. Finally, we applied untargeted metabolomics based on ultra-performance liquid chromatography-quadrupole time-of-flight mass spectrometry (UPLC-QTOF) to systematically characterize metabolic changes and investigate their associations with the preventive effects of ES.

## 2. Materials and Methods

### 2.1. Extraction, Fractionation, and Identification of ES

ES was collected from Rizhao, Shandong Province, China, and authenticated by Prof. Zuntian Zhao, Shandong Normal University. A voucher specimen was deposited at Qingdao University. The whole plant was dried at room temperature, pulverized, and extracted with 95% ethanol using intermittent ultrasonic oscillation for 8 h. The solvent was removed under vacuum to yield the whole extract of ES. The extract was then partitioned twice between ethyl acetate and distilled water. The ethyl acetate extract was further separated by column chromatography using Sephadex LH-20 (GE Healthcare, Chicago, IL, USA). Methanol was used as the elution solvent, and six fractions were collected from the resulting eluate. Each fraction was further recrystallized from methanol to afford fractions F1–F6. The extraction and fractionation process was summarized in Supplementary Figure S1.

The structure of F5 was assigned using ^1^H NMR and ^13^C NMR recorded at 400 and 100 MHz, respectively, on a JNM-ECZ600R spectrometer (JEOL Ltd., Akishima, Japan). Measurements were performed in dimethylsulfoxide (DMSO)-d6, with tetramethylsilane as the chemical-shift reference. Ethanol, ethyl acetate, methanol, and DMSO-d6 were purchased from Shanghai Hushi Laboratory Equipment Co., Ltd. (Shanghai, China). Sephadex LH-20 was purchased from GE Healthcare (USA).

### 2.2. In Vitro Evaluation for Antibacterial and Antioxidant Properties

The reference bacterial strains used for antibacterial evaluation, *Escherichia coli* and *Staphylococcus aureus*, were sourced from the American Type Culture Collection (ATCC, Manassas, VA, USA). Antibacterial activity against these strains was determined for ES and F1-F6 using the agar diffusion assay. Each sample was prepared in DMSO to a concentration of 2 mg/mL, and a 70 μL aliquot was loaded into an Oxford cup placed on Petri dishes containing *E. coli* or *S. aureus*. DMSO was used as the negative control. Inhibition zones were recorded after incubation at 37 °C for 12 h.

Free-radical scavenging capacity was evaluated with 2,2-diphenyl-1-picrylhydrazyl (DPPH). For each reaction, a 100 μL aliquot of sample was combined with 100 μL DPPH reagent prepared in ethanol at 0.2 mg/mL. The reaction system was protected from light and maintained at 37 °C for 30 min. Residual DPPH was then quantified spectrophotometrically at 517 nm, and the half-maximal inhibitory concentration was derived from the corresponding scavenging activity.

### 2.3. In Vitro Evaluation for Anti-Inflammatory Property

RAW 264.7 cells were supplied by the Chinese National Collection of Authenticated Cell Cultures. The complete culture medium consisted of Dulbecco’s Modified Eagle’s Medium (Shanghai Vivacell Biosciences Ltd., Shanghai, China), 10% fetal bovine serum (Pricella Biotechnology Co., Ltd., Wuhan, China), and 1% penicillin-streptomycin (BasalMedia Technologies Co., Ltd., Wuhan, China). Cells were maintained in a humidified incubator set at 37 °C and 5% CO_2_.

For the cell viability assay, ES, F5, and F6 were prepared at 0, 25, 50, 100, 200, 400, 800, and 1600 μg/mL and applied to RAW 264.7 cells for 24 h. After exposure, cell viability was determined using the Cell Counting Kit-8 assay (TargetMol Chemicals Inc., Wellesley Hills, MA, USA) in accordance with the manufacturer’s protocol.

For the anti-inflammatory assay, cells were assigned to five groups: control (CON), lipopolysaccharide (LPS)-stimulated inflammatory (Model), F5, F6, and ES treatment groups. Except for the CON group, cells were stimulated with 1 μg/mL LPS (Sigma-Aldrich, St. Louis, MO, USA) for 24 h. The doses of ES, F5, and F6 were 25 μg/mL, 100 μg/mL, and 100 μg/mL, respectively. After incubation, cellular morphology was examined under a microscope. Culture supernatants were harvested, and the concentrations of TNF-α and interleukin-6 (IL-6) were measured using enzyme-linked immunosorbent assay (ELISA) kits (Jingmei Biotechnology Co., Ltd., Yancheng, China).

### 2.4. In Vivo Animal Experiments

Ethical approval for the animal experiment was granted by the Experimental Animal Welfare Ethics Committee of Qingdao University (No. 20240823DBA/1JGpt7020241025209). All animal-related procedures were performed following the US National Research Council’s Guide for the Care and Use of Laboratory Animals [13].

Forty-five male DBA/1JGpt mice at 8 weeks’ age were obtained from Jiangsu GemPharmatech Co., Ltd. (Nanjing, China). Before the experiment, the animals were kept in a specific pathogen-free animal facility, where the temperature was controlled at 20–26 °C, the relative humidity was maintained at 40–70%, and a 12 h light/12 h dark cycle was applied. Standard food and water were available ad libitum throughout the study.

Mice were allowed to acclimate for 7 days before being randomized into five groups (n = 9 for each group): control (CON), model, low-dose extract (ES-L), high-dose extract (ES-H), and ellagic acid group. The randomization was conducted using a random number table. The nine mice in each group were kept in two cages. The cage positions were counterbalanced across groups, and no cage rotation was conducted to avoid stress.

The sample size was referred to previous literature [8,14,15]. Although a prospective power calculation was not performed, we conducted a post hoc sensitivity analysis to evaluate sample size adequacy. Based on a previous study [16] reporting a mean difference of 0.66 mm in paw thickness and a pooled standard deviation of 0.34 mm, the minimum required sample size was 6 mice per group (PASS 15.0 software, two-tailed *t*-test, α = 0.05, power = 0.80). Our actual sample size (n = 9 per group) exceeded this requirement, with the additional mice allocated to account for potential attrition over the experiment.

The model was established according to previous literature [17]. For primary immunization, bovine type II collagen (Shanghai Yuanye BioTechnology Co., Ltd., Shanghai, China) was dissolved in 10 mM acetic acid to obtain a 4 mg/mL collagen solution. The collagen solution was then combined with an equal volume of complete Freund’s adjuvant (Sigma-Aldrich, USA) to prepare the primary emulsion. On day 21, booster immunization was performed with an emulsion containing bovine type II collagen (2 mg/mL) and an equal volume of incomplete Freund’s adjuvant (Sigma-Aldrich, USA). Each mouse received 100 μL emulsion by subcutaneous injection at the base of the tail. The immunizations were performed in the model, ES-L, ES-H, and ellagic acid groups.

ES-L, ES-H, and ellagic acid were received at daily doses of 375, 750, and 250 mg/kg body weight, respectively. The dose of ES was derived from the *Chinese Pharmacopoeia* (9–15 g/60 kg adult) via body surface area conversion (1.8–3 g/kg in mice), but the theoretical dose was reduced to 750 mg/kg after preliminary experiments showed that it caused weight loss. This adjusted dose remained well below the reported no-observed-adverse-effect level (>4500 mg/kg) for related species [18], and endpoint histopathology confirmed no organ toxicity. All test compounds were dissolved in 0.5% sodium carboxymethylcellulose (Shanghai Macklin Biochemical Co., Ltd., Shanghai, China) and administered by oral gavage once daily for 8 weeks. The model and CON received equal amounts of 0.5% sodium carboxymethylcellulose. The intervention started on the first day of immunization.

Two mice were excluded from the final analysis due to procedure-related fatalities. One mouse from the ES-L group was lost due to restraint-induced respiratory failure during booster immunization, and one mouse from the CON group died from idiopathic gastric dilatation. Throughout the intervention period, body weight, paw thickness, and arthritis scores were monitored on a three-time-weekly schedule. The order of treatments and measurements was randomized using a sequence generated by a random number table. The outcome assessor and data analyzer were blinded to the group allocation.

At the end of the study, the mice were euthanized, after which ankle joint tissues and blood were collected for subsequent analyses. Serum was prepared from the collected blood by centrifugation at 3500 rpm for 15 min at 4 °C.

### 2.5. Assessment of Paw Thickness and Arthritis Severity

Paw thickness was determined with a vernier caliper. Arthritis severity was scored separately for each paw using a 0–4 scale [19]. A score of 0 indicated no visible erythema or swelling; 1 indicated mild erythema and swelling restricted to the tarsal region or ankle joint; 2 indicated mild erythema and swelling extending from the ankle to the tarsals; 3 indicated moderate erythema and swelling extending from the ankle to the metatarsal joints; and 4 indicated severe erythema and swelling involving the ankle, foot, and digits, or ankylosis of the limb. The scores from the four paws were added to obtain the total arthritis score for each mouse, with a maximum score of 16.

### 2.6. Measurement of TNF-α and IL-6 and Histological Analysis of Ankle Joints

Serum TNF-α and IL-6 concentrations were determined using ELISA kits (Jingmei Biotechnology Co., Ltd., China) in accordance with the manufacturer’s instructions. Six mice in each group were subject to histological analysis due to budget constraints. The six mice were selected by proportionate stratified random sampling according to predefined arthritis score level (low, 0–1; medium, 1–4; and high, ≥4). The proportion of selected mice in each stratum matched that of the original group. Randomization in each stratum was performed using a random number table. Ankle joint tissues were processed for histological evaluation by sequential fixation in neutral formalin solution, decalcification, dehydration, and paraffin embedding. The prepared paraffin sections were then examined using hematoxylin and eosin (H&E) staining together with Safranin O/Fast Green staining. Based on previously described criteria [20], four histopathological parameters were evaluated: inflammatory cell infiltration, bone erosion, cartilage damage, and proteoglycan loss. Each parameter was graded on a 0–3 scale, with 0 representing normal and 3 representing severe. The total histopathology score was obtained by adding the four individual scores, with 12 as the highest possible value.

### 2.7. Serum Metabolite Profiling by UPLC-QTOF

The same six mice in each group subject to histological analysis were analyzed for serum metabolites due to budget constraints. Serum samples were prepared by adding pre-cooled acetonitrile and keeping the mixtures on ice for 15 min. The treated samples were centrifuged at 15,000 rpm for 15 min, after which the supernatant was collected for subsequent metabolomic analysis. Metabolite separation and detection were carried out using UPLC-QTOF (Agilent Technologies, Inc., Santa Clara, CA, USA) equipped with an ACQUITY UPLC BEH C18 column (100 mm × 2.1 mm, 1.7 µm). For each run, a 5 μL sample was injected, and chromatographic separation was performed at a flow rate of 0.4 mL/min. The mobile phase system consisted of solvent A, acetonitrile containing 0.1% formic acid, and solvent B, water containing 0.1% formic acid. The capillary voltage was set at 4500 V. Full-scan mass spectra were collected across an m/z range of 50–1000. The acquisition rate was set to 2 spectra/s, with an acquisition time of 500 ms/spectrum. During detection, the drying gas temperature was maintained at 350 °C, and the drying gas flow rate was 8 L/min.

Data were processed with MS-DIAL (version 4.38, University of California, Davis, USA; RIKEN CSRS, Japan) for peak detection, noise reduction, alignment, and normalization. MetaboAnalyst 5.0 (http://www.metaboanalyst.ca) was used for differential metabolite and metabolic pathway analyses and heatmap construction. Metabolite identification was conducted by searching against the Human Metabolome Database. Principal coordinate analysis (PCoA) was carried out using Bray–Curtis dissimilarity. For multiple testing correction, the Benjamini–Hochberg procedure was used to control the false discovery rate.

### 2.8. Statistical Analysis

Data were shown as mean ± standard error. For comparisons among groups, one-way analysis of variance was used, with Tukey’s post hoc test applied for subsequent multiple comparisons. Metabolite-disease severity relationships were examined by Spearman’s correlation analysis. Statistical processing was conducted using SPSS 26.0 software (IBM, Armonk, NY, USA). A threshold of *p* < 0.05 was used to define statistical significance.

## 3. Results

### 3.1. Antibacterial, Antioxidant, and Anti-Inflammatory Activities of ES-Derived Polyphenols

Among all fractions of ES, F5 showed the most pronounced inhibitory effects on both *E. coli* and *S. aureus* (Supplementary Figure S2). F6 displayed an inhibitory effect against *S. aureus* comparable to that of F5 but was less effective against *E. coli* than F5. F3 and F4 showed moderate inhibitory effects, whereas F1 and F2 barely exhibited any antibacterial activity. The antioxidant activities of F5 and F6 were next evaluated using the DPPH radical scavenging assay. Both fractions exhibited concentration-dependent scavenging activity (Supplementary Figure S3).

**Figure 1:**
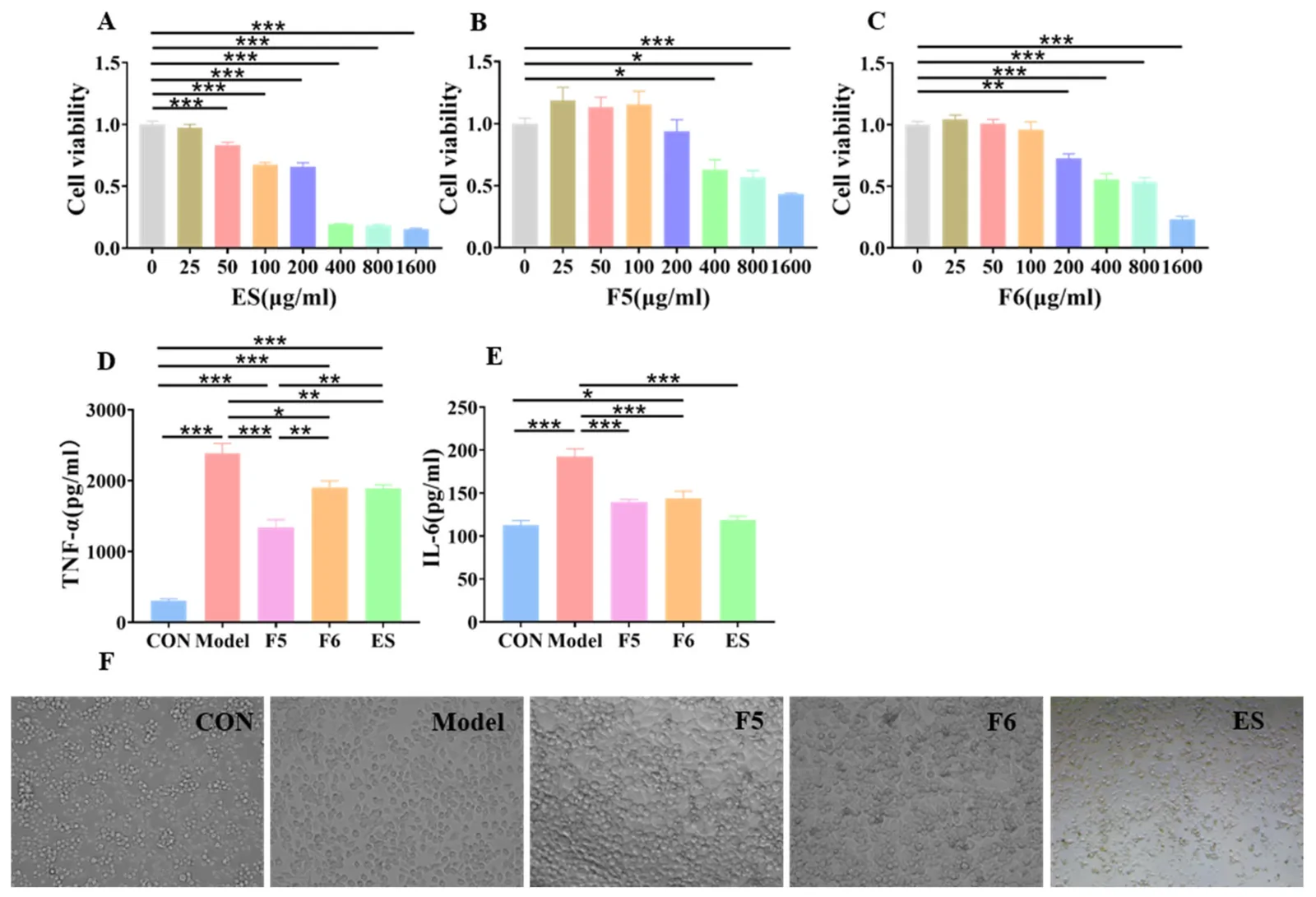
Anti-inflammatory effects of ES, F5, and F6. (**A**–**C**) RAW 264.7 cells were incubated with serial doses of ES (**A**), F5 (**B**), or F6 (**C**) from 0 to 1600 μg/mL, followed by assessment of cell viability. (**D**,**E**) TNF-α (**D**) and IL-6 (**E**) released into the culture medium were measured in the CON, Model, F5, F6, and ES groups. (**F**) Representative images showing RAW 264.7 cell morphology under the indicated treatments. CON, control group; Model, lipopolysaccharide-stimulated group; F5, fraction 5 group; F6, fraction 6 group; ES, whole *E. stephanianum* extract group. Results are reported as the mean ± standard error. Statistical comparisons were performed using one-way ANOVA with Tukey’s multiple-comparison test. *, **, and *** indicate statistical significance at *p* < 0.05, *p* < 0.01, and *p* < 0.001, respectively.

The anti-inflammatory activities of ES, F5, and F6 were subsequently evaluated using RAW 264.7 macrophages stimulated with LPS (Figure 1). Cell viability assays showed that ES, F5, and F6 caused no significant cytotoxic effects at concentrations up to 25, 100, and 100 μg/mL, respectively (Figure 1A–C). These concentrations were selected for the following experiments. Compared with the CON group, LPS stimulation led to a significant increase in TNF-α and IL-6 secretion by RAW 264.7 cells (Figure 1D,E). Compared with the LPS group, ES, F5, and F6 reduced the levels of both cytokines, among which F5 showed the strongest inhibitory effect. Microscopic observation also showed that LPS stimulation caused obvious inflammatory morphological alterations in RAW 264.7 cells, whereas these alterations were partly alleviated after treatment with ES, F5, or F6 (Figure 1F).

**Figure 2:**
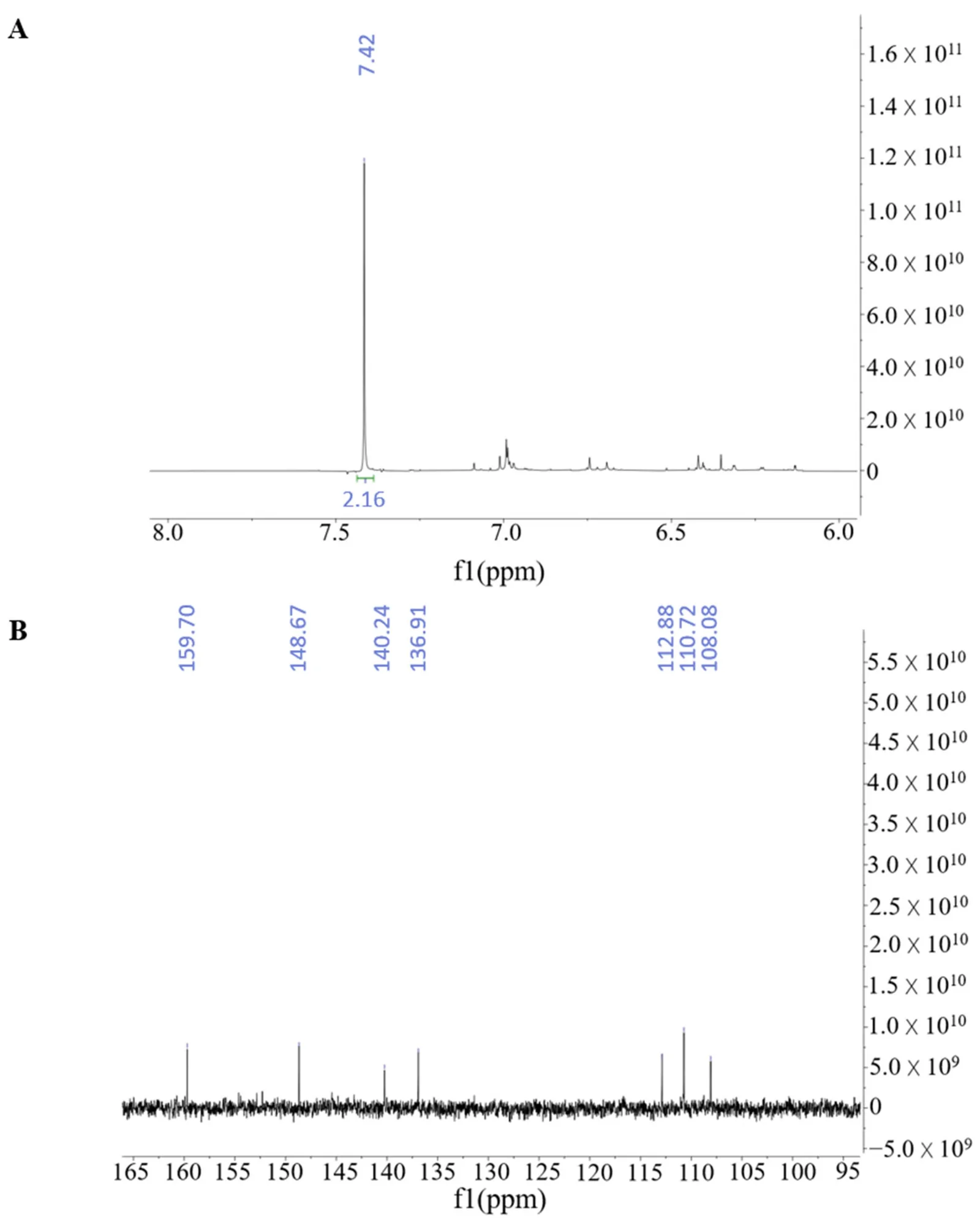
(**A**) ^1^H NMR spectrum of F5. (**B**) ^13^C NMR spectrum of F5. F5, fraction 5 group.

Based on its consistently stronger antibacterial, antioxidant, and anti-inflammatory activities, F5 was chosen for structural characterization and further in vivo evaluation. The structural assignment indicated that F5 corresponded to ellagic acid, as supported by its ^1^H NMR and ^13^C NMR spectra (Figure 2). The observed chemical shift data were in good agreement with those previously reported for ellagic acid [21].

### 3.2. ES-Derived Polyphenols Prevented CIA Progression in Mice

**Figure 3:**
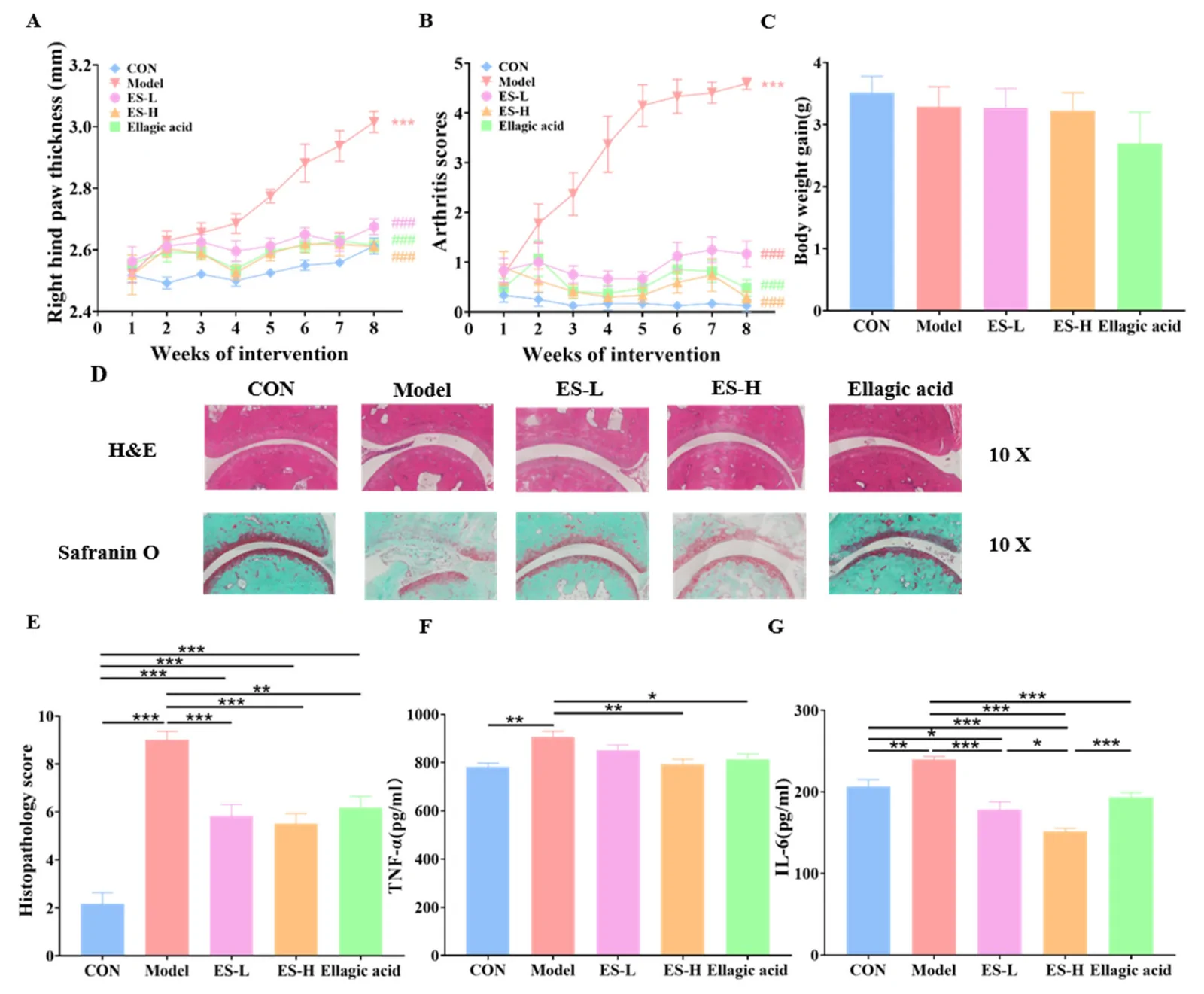
(**A**) Right hind paw thickness. (**B**) Arthritis scores. (**C**) Body weight gain after 8 weeks’ intervention. (**D**) Representative histological images of ankle joints stained with H&E and Safranin O/Fast Green (10× magnification). (**E**) Histopathology score. (**F**) Serum TNF-α level. (**G**) Serum IL-6 level. CON, normal control group; model, collagen-induced arthritis group; ES-L, low-dose *E. stephanianum* extract group; ES-H, high-dose *E. stephanianum* extract group. Data are reported as the mean ± standard error. For panels (**A**,**B**), statistical comparisons were performed by repeated measures ANOVA, followed by Tukey’s HSD post hoc tests for pairwise comparisons; for panels (**C**,**E**–**G**), statistical comparisons were performed using one-way ANOVA with Tukey’s multiple-comparison test. For clarity, only the statistical significance at the final week is shown in (**A**,**B**). For panels (**A**–**C**), * and # denote significance versus CON and versus model, respectively. *, **, *** and represent *p* < 0.05, 0.01, and 0.001, respectively. ### represents *p* < 0.001. For panels (**A**–**C**,**F**,**G**), n = 9 per group (except that n = 8 for the CON and ES-L group; mice died due to procedure-related fatalities). For panels (**D**,**E**), n = 6 per group.

In the final week, arthritis scores and right hind paw thickness were markedly elevated in the model group compared with the CON group. Each of the three interventions significantly decreased these two parameters, while no significant differences were detected among the treatment groups (Figure 3A,B). For example, the effect sizes (95% CI) of ES-H vs. model for arthritis score and right hind paw thickness were −4.30 (−4.94, −3.65), *p* < 0.001 and −0.4 mm (−0.5 mm, −0.3 mm), *p* < 0.001, respectively. After intervention, body weight gain remained comparable across all groups (Figure 3C).

Figure 3D presents representative ankle joint sections stained with H&E and Safranin O/Fast Green. Compared with the CON group, the model group exhibited more severe histopathological lesions, as evidenced by prominent inflammatory cell accumulation, bone erosive changes, proteoglycan depletion, and cartilage injury in the ankle joints, together with a markedly elevated histopathological score (Figure 3D,E). Treatment with ES-H, ES-L, or ellagic acid significantly prevented these changes and reduced the histopathological score compared with the model group, while the preventive effects were comparable among the three intervention groups.

The model group showed markedly higher serum concentrations of TNF-α and IL-6 than the CON group (Figure 3F,G). The ES-H and ellagic acid groups had significantly reduced TNF-α levels, whereas ES-L treatment had no significant effect. All three treatment groups had significantly reduced IL-6 levels, with ES-H showing a superior effect compared to ES-L and ellagic acid (Figure 3F,G).

### 3.3. ES-Derived Polyphenols Altered Serum Metabolite Profiles in CIA Mice

**Figure 4:**
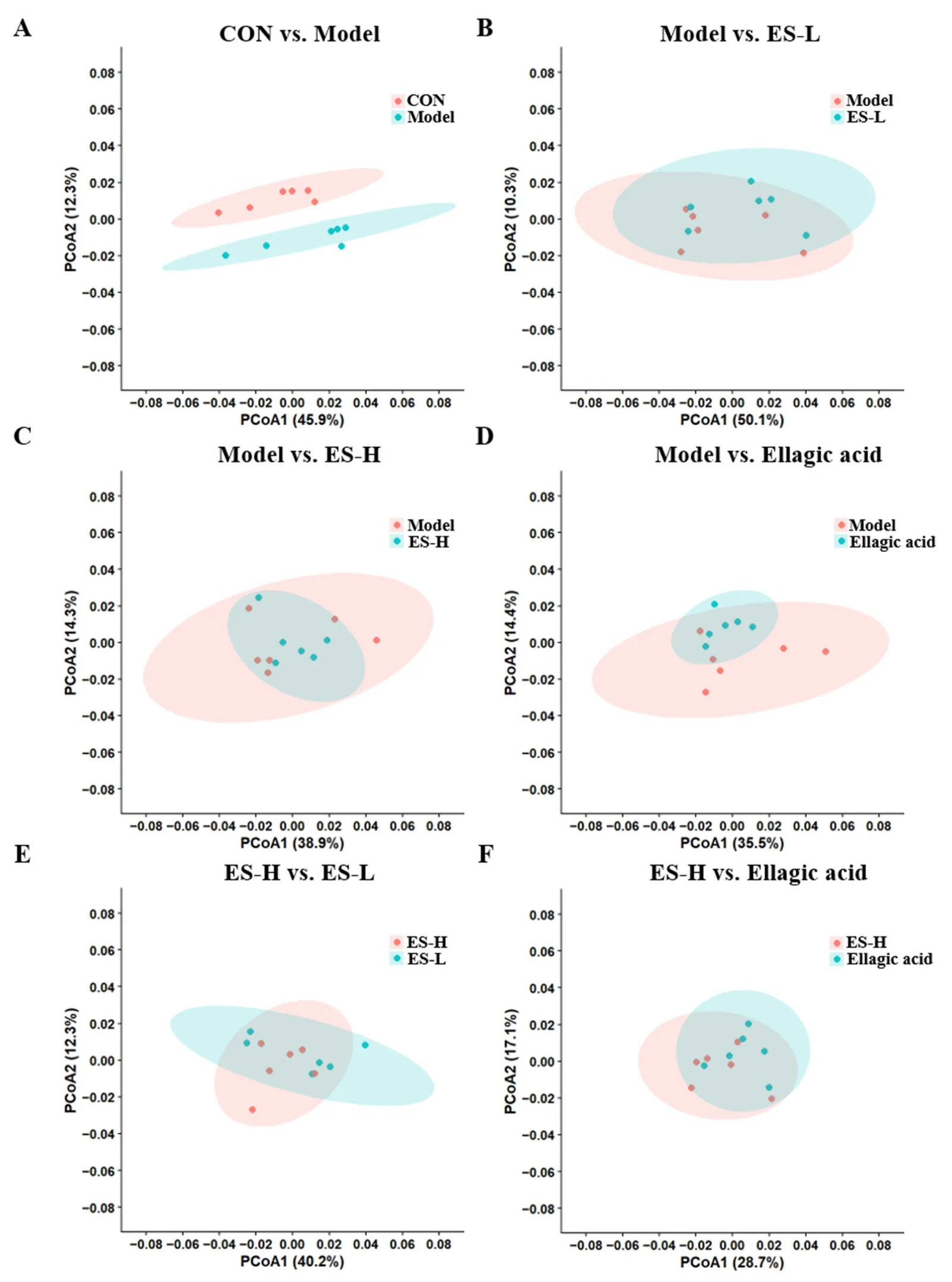
Principal coordinate analysis (PCoA) plots of serum metabolomic profiles based on Bray–Curtis dissimilarity. (**A**) CON vs. Model; (**B**) Model vs. ES-L; (**C**) Model vs. ES-H; (**D**) Model vs. Ellagic acid; (**E**) ES-H vs. ES-L; and (**F**) ES-H vs. Ellagic acid. CON, normal control group; model, collagen-induced arthritis group; ES-L, low-dose *E. stephanianum* extract group; ES-H, high-dose *E. stephanianum* extract group. N = 6 per group.

Pairwise PCoA analysis was performed to compare serum metabolomic profiles among groups (Figure 4). A clear separation was observed between the CON and model groups.

**Figure 5:**
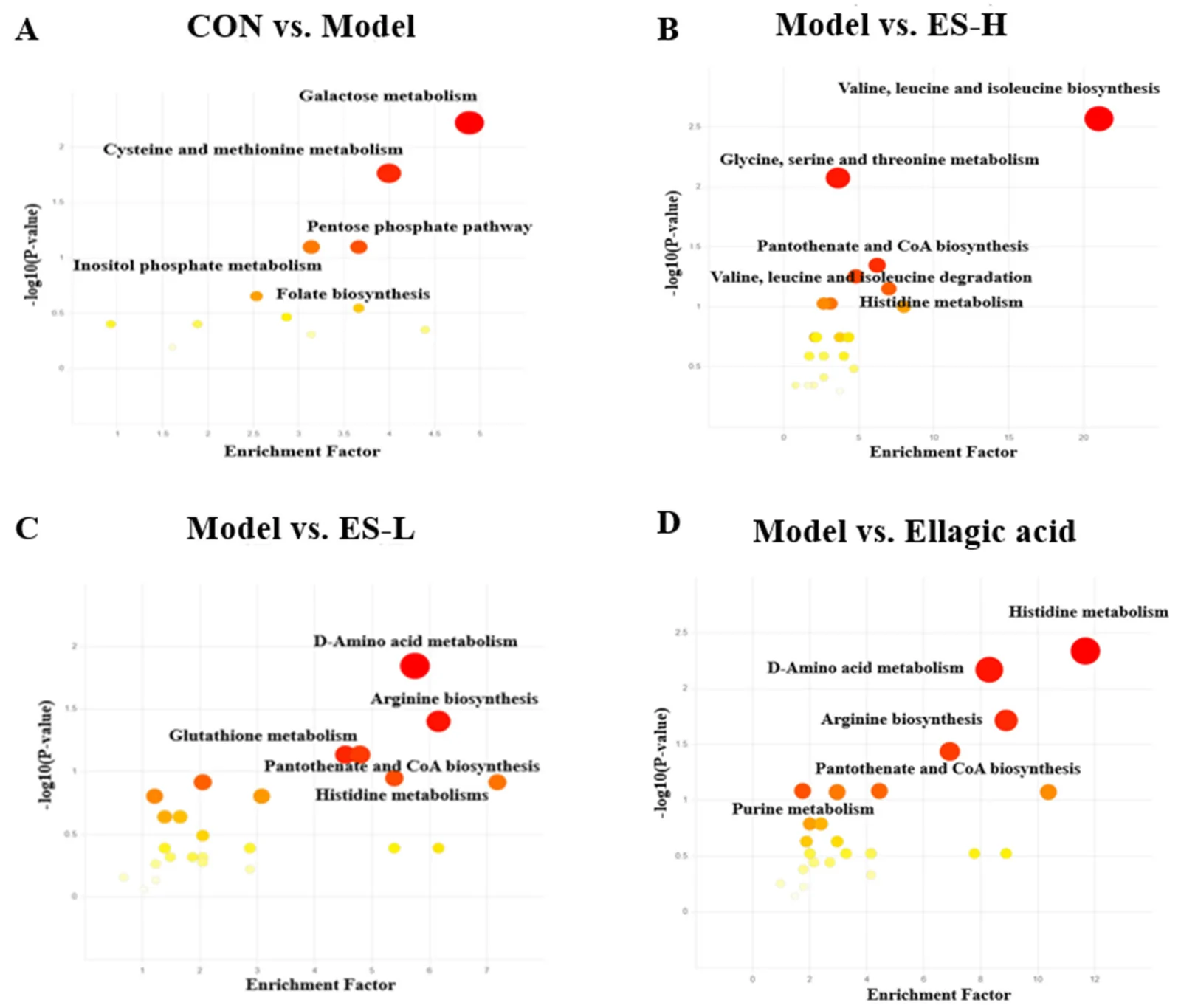
KEGG pathway enrichment analysis across group comparisons. (**A**) CON vs. Model; (**B**) Model vs. ES-H; (**C**) Model vs. ES-L; and (**D**) Model vs. Ellagic acid. CON, normal control group; model, collagen-induced arthritis group; ES-L, low-dose *E. stephanianum* extract group; ES-H, high-dose *E. stephanianum* extract group. N = 6 per group.

Differential metabolic pathways across groups were explored using KEGG pathway analysis. The major differential pathways between the CON and model groups were galactose metabolism and cysteine and methionine metabolism (Figure 5A; Supplementary Table S1). In the model versus ES-H comparison, the principal altered pathways included valine, leucine, and isoleucine biosynthesis; glycine, serine and threonine metabolism; and pantothenate and coenzyme A (CoA) biosynthesis (Figure 5B; Supplementary Table S2). For the model versus ES-L comparison, the principal altered pathways included D-amino acid metabolism and arginine biosynthesis (Figure 5C; Supplementary Table S3). Between the model and ellagic acid groups, the major differential pathways were histidine metabolism, D-amino acid metabolism, arginine biosynthesis, and pantothenate and CoA biosynthesis (Figure 5D; Supplementary Table S4).

**Figure 6:**
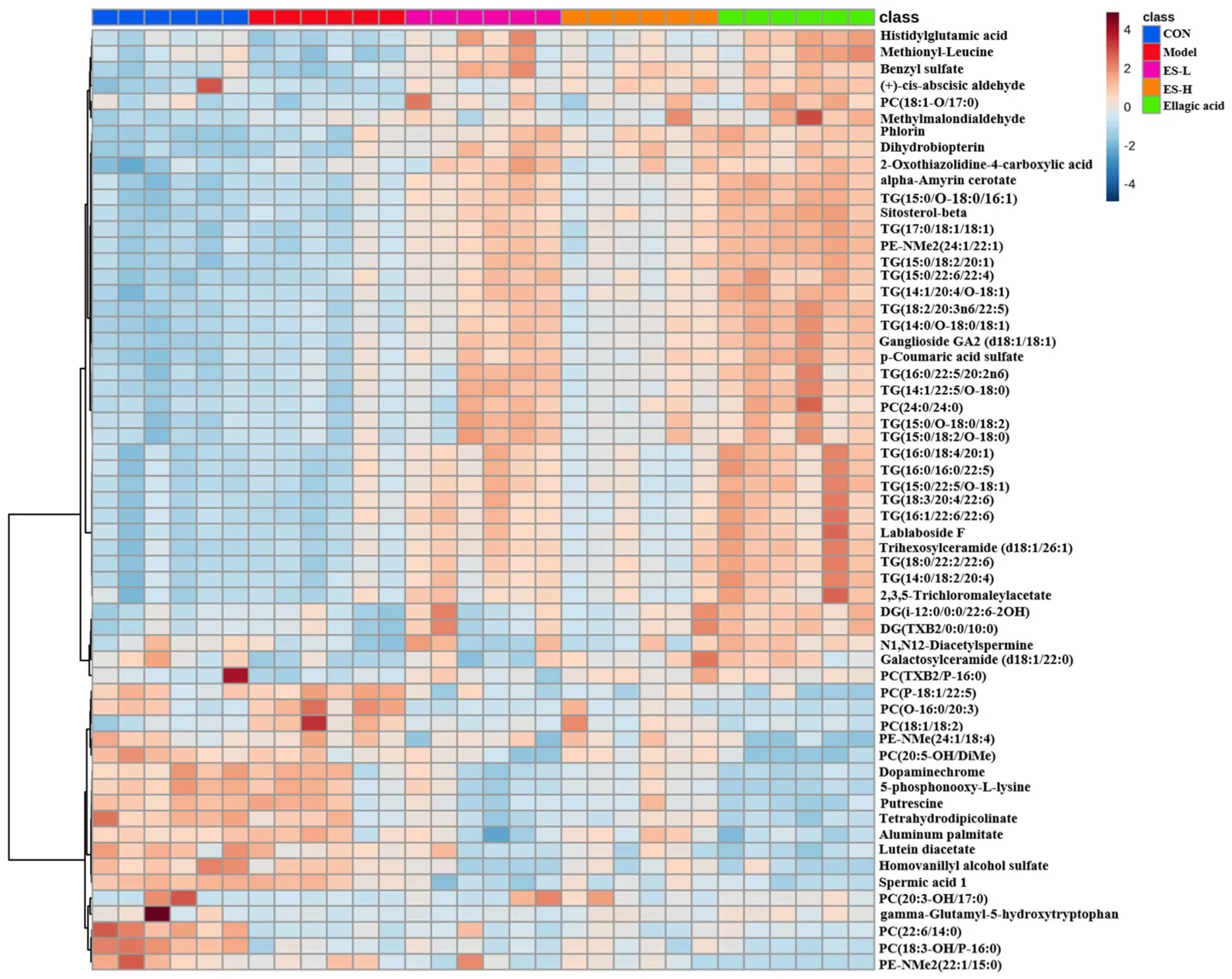
Hierarchical clustering of differential serum metabolites among the five groups (n = 6 per group). The normalized relative abundance of metabolites is represented by the color scale, with lower and higher abundance shown in blue and red, respectively. CON, normal control group; model, collagen-induced arthritis group; ES-L, low-dose *E. stephanianum* extract group; ES-H, high-dose *E. stephanianum* extract group.

Across the five groups, 59 differential metabolites were identified (Figure 6; Supplementary Tables S5–S8). These metabolites were further classified into three distinct clusters by hierarchical cluster analysis according to their alteration patterns. Cluster one consisted of 41 metabolites, which showed an upward trend in the ES-L, ES-H, and ellagic acid groups compared to the model group. Cluster two comprised 13 metabolites that exhibited a downward trend in the ES-L, ES-H, and ellagic acid groups compared to the model group. Cluster three contained five metabolites that decreased in the model, ES-L, ES-H, and ellagic acid groups compared with the CON group.

### 3.4. Associations Between Differential Serum Metabolites and Disease Severity

**Figure 7:**
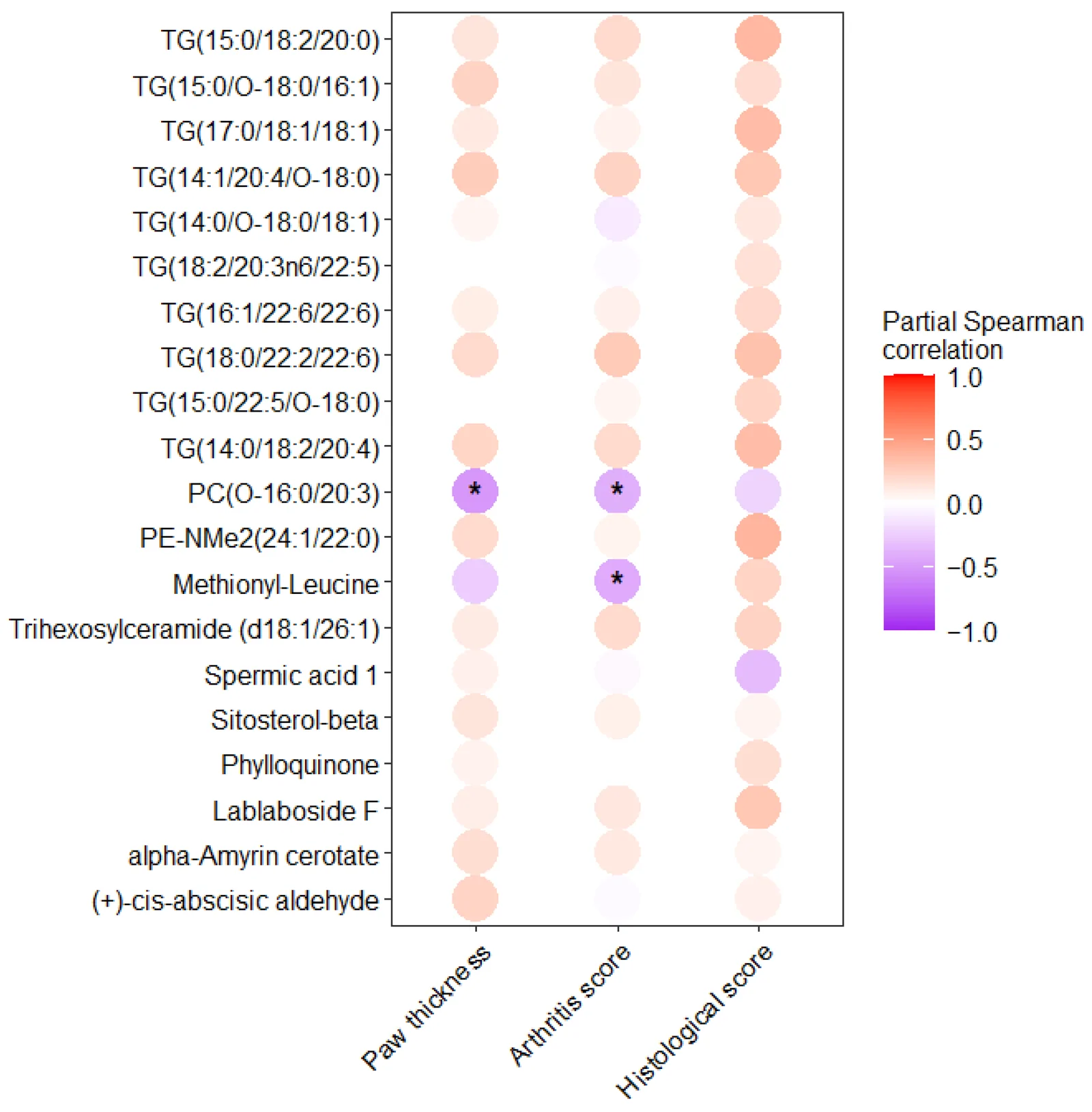
Spearman rank partial correlation analysis (adjusting group allocation as covariate) of differential serum metabolites with paw thickness, arthritis score, and histological score. Correlation polarity is color-coded, with positive and negative associations shown in red and purple, respectively. * indicates statistical significance at *p* < 0.05. N = 6 per group.

Associations of differential metabolites with disease severity indicators, including right hind paw thickness, arthritis score, and histological score, were assessed using Spearman rank partial correlation analysis, which adjusted group allocation as a covariate (Figure 7).

Methionyl-leucine showed a negative correlation with arthritis score, and PC(O-16:0/20:3) showed negative correlations with paw thickness and arthritis score. The associations between other metabolites and disease severity were not significant.

## 4. Discussion

This study showed that ES-derived polyphenols, like ellagic acid, displayed potent anti-inflammatory, antibacterial, and antioxidant activities in vitro. In animal experiments, ES-derived polyphenols prevented the progression of arthritic phenotypes and decreased pro-inflammatory cytokine levels. Serum metabolomic analysis further revealed that the protective effects of ES-derived polyphenols were associated with the remodeling of multiple metabolic pathways, including lipid metabolism (e.g., triacylglycerols and phospholipids), amino acid metabolism, and purine metabolism.

In vitro assays revealed pronounced anti-inflammatory, antibacterial, and antioxidant activities of ES-derived polyphenols. In line with these findings, previous studies reported that ES attenuated LPS-induced inflammatory mediator production in macrophages by suppressing NF-κB and MAPK signaling, as reflected by reduced levels of nitric oxide (NO) and pro-inflammatory cytokines, including TNF-α, IL-1β, and IL-6 [22]. In terms of antioxidant activity, ES is rich in tannins, flavonoids, and phenolic acids, which can efficiently scavenge free radicals and provide antioxidant protection [23]. Furthermore, the extract of this plant disrupts microbial cell membrane integrity, inhibits biofilm formation, and interferes with nucleic acid synthesis, thereby demonstrating broad-spectrum antibacterial activity [24,25].

Ellagic acid (F5) exhibited an in vivo effect on preventing CIA progression comparable to ES extract, indicating that ellagic acid is an important contributor to the preventive effect of ES extract on CIA. Consistent with this, earlier studies reported that ellagic acid attenuated paw swelling, disease severity, and synovial lesions in CIA rats, while also providing cartilage- and joint-protective effects in adjuvant-induced arthritis [14,15,16]. Mechanistically, these effects have been attributed to suppression of the NF-κB cascade and inhibition of pro-inflammatory cytokines [15,16]. That said, the contributions of the other constituents of ES extract to the effect on preventing CIA progression cannot be ruled out.

Serum metabolomic analyses indicated that ES-derived polyphenols prevented CIA progression in mice via several classes of serum metabolites. First, several triacylglycerols may mediate the preventive effect, including TG(15:0/18:2/20:1), TG(15:0/O-18:0/16:1), TG(17:0/18:1/18:1), TG(14:1/20:4/O-18:1), TG(14:0/O-18:0/18:1), TG(18:2/20:3n6/22:5), TG(16:1/22:6/22:6), TG(18:0/22:2/22:6), TG(15:0/22:5/O-18:1), and TG(14:0/18:2/20:4). These species were elevated after intervention. Triacylglycerols have been shown to increase in patients with a good treatment response of RA and to be associated with improvement in disease activity [26].

Notably, several of the altered triacylglycerol species contained polyunsaturated fatty acid side chains with immunomodulatory properties. Specifically, TG(18:2/20:3n6/22:5) and TG(15:0/22:5/O-18:1) contained docosapentaenoic acid (DPA), whereas TG(16:1/22:6/22:6) and TG(18:0/22:2/22:6) contained docosahexaenoic acid (DHA). Both DHA and DPA are *n*-3 polyunsaturated fatty acids, which were shown to downregulate pro-inflammatory cytokine production and protect against cartilage degradation [27]. A previous meta-analysis showed that fish oil supplementation (rich in DHA and DPA) reduced morning stiffness, joint swelling, and pain in RA patients, and decreased the required doses of glucocorticoids [28]. Triacylglycerols esterified with DHA and DPA may act as a “fatty acid buffer pool”, and they can be released via lipolysis to exert anti-inflammatory effects [29]. Additionally, TG(15:0/18:2/20:1) and TG(14:0/18:2/20:4) contained linoleic acid (LA) side chains. Compared with healthy controls, RA patients have been reported to exhibit lower LA levels, and this reduction in LA has been associated with increased disease severity [30].

Second, three additional metabolites may also mediate the preventive effects of ES-derived polyphenols. Methionyl-leucine, a dipeptide, was elevated following interventions and negatively associated with disease severity. Methionyl-leucine may exert antioxidant and immunomodulatory effects: methionine scavenges reactive oxygen species, while leucine modulates mechanistic target of rapamycin (mTOR) signaling [31]. Trihexosylceramide (d18:1/26:1), a glycosphingolipid, was increased following interventions. Glycosphingolipids are key components of lipid rafts and regulate immune cell signaling; their metabolism has been linked to inflammation resolution and T-cell regulation [32]. In contrast, Spermic acid 1 (N(8)-2-carboxyethylspermidine), a spermidine derivative, was suppressed following treatment. Spermidine is a polyamine known to be elevated in active RA and to promote T-cell proliferation, pro-inflammatory cytokine production, and osteoclastogenesis [33,34,35]. The decrease in this spermidine derivative after intervention may contribute to the resolution of joint inflammation.

KEGG pathway analyses indicated that several metabolic pathways played important roles in mediating the effect of ES-derived polyphenols on preventing CIA in mice. First, amino acid metabolism was prominently affected, including the metabolism of valine, leucine, isoleucine, glycine, serine, threonine, histidine, arginine, glutathione, and D-amino acids. These pathways are closely linked to the pathogenesis of RA [11,36]. Branched-chain amino acids (valine, leucine, isoleucine) activated mTOR signaling, promoting T-cell and synovial fibroblast proliferation and exacerbating joint inflammation [36]. Glycine exerted anti-inflammatory effects by suppressing TNF-α and IL-6 production, and its levels are often reduced in RA [37]. Serine and threonine contributed to glutathione synthesis and membrane integrity [36]. Histidine served as a precursor of histamine, a vasoactive mediator that aggravates synovitis [38]. Arginine metabolism generated NO and polyamines, both of which promoted inflammation and bone erosion [39]. D-amino acids, derived from gut microbiota or racemization, may modulate immune responses and *N*-methyl-D-aspartate receptor signaling [40]. Glutathione metabolism is a major antioxidant system. Depletion of glutathione exacerbated reactive oxygen species accumulation and oxidative stress, a key driver of synovial inflammation and joint destruction in RA [10]. The coordinated modulation of these pathways by ES preparations may contribute to its immunoregulatory and antioxidant effects, reflecting the restoration of metabolic homeostasis in RA.

Purine metabolism is intimately connected to adenosine signaling, and adenosine serves as an endogenous anti-inflammatory mediator that suppresses T-cell activation and reduces pro-inflammatory cytokine production [41]. In RA, increased adenosine deaminase activity accelerates the degradation of adenosine and thereby promotes synovial inflammation [41]. By modulating the purine metabolism pathway, ES preparations may restore adenosine levels and strengthen adenosine-mediated anti-inflammatory signaling.

Additionally, pantothenate and CoA biosynthesis were also significantly altered. CoA is essential for mitochondrial energy production, fatty acid β-oxidation, and lipid synthesis. Activated synovial fibroblasts and immune cells in RA have high metabolic demands [42]. Changes in CoA availability may affect energy homeostasis and the generation of pro-inflammatory lipids, such as prostaglandins and leukotrienes. The regulatory effect of ES preparations on this pathway suggests that improved energy homeostasis may contribute to their preventive effects on CIA [43].

That said, the present metabolomic results only showed associations of these potential metabolites and metabolic pathways with effects on preventing CIA progression. The causal relationships need to be established by future molecular experiments.

A few limitations need to be acknowledged. First, the in vitro LPS-macrophage screen reflects innate immunity, whereas CIA is adaptive autoimmune. This assay was used solely for preliminary candidate filtering; preventive efficacy was confirmed in the CIA model. Second, only male DBA/1JGpt mice were used, which may limit the generalizability to females. Third, although collagen-induced arthritis is the most common model in the literature, it is not identical to rheumatoid arthritis in humans. A future randomized controlled trial is needed to validate the effects of ES-derived polyphenols on human rheumatoid arthritis. Fourth, a positive control was not used. Future work needs to include a positive control to demonstrate the clinical relevance of the observed effect. Fifth, the metabolomic observations were exploratory due to the reduced number of mice and lack of validation using reference standards. Sixth, further characterization of the compounds in the ES extract needed to be performed in future research.

## 5. Conclusions

ES-derived polyphenols, like ellagic acid, prevented CIA progression in mice, which was associated with their modulations on serum metabolites, including increased anti-inflammatory triacylglycerols and amino acid and purine metabolism. These results supported further investigation of ES-derived ellagic acid in preventing RA in a human randomized controlled trial.

## Data Availability

Data will be made available on reasonable request.

## Supplementary Materials

The following supporting information can be downloaded at: https://www.mdpi.com/article/10.3390/nu18162683/s1, Figure S1: Flowchart illustrating the extraction and fractionation of Erodium stephanianum; Figure S2: Antibacterial activities of the whole extract of Erodium stephanianum (ES) and its fractions (F1–F6) against Escherichia coli (A) and Staphylococcus aureus (B); Figure S3: Antioxidant activities of fractions 5 (F5) and 6 (F6) from the Erodium stephanianum extract. Table S1: Differential metabolic pathways between the CON and Model groups; Table S2: Differential metabolic pathways between the Model and ES-H groups; Table S3: Differential metabolic pathways between the Model and ES-L groups; Table S4: Differential metabolic pathways between the Model and Ellagic acid groups; Table S5: The differential metabolites between the CON and Model groups in the serum; Table S6: The differential metabolites between the ES-L and Model groups in the serum; Table S7: The differential metabolites between the Ellagic acid and Model groups in the serum; Table S8: The differential metabolites between the ES-H and Ellagic acid groups in the serum.
