# Supplementary material for: Erodium stephanianum-Derived Polyphenols Prevented the Progression of Collagen-Induced Arthritis in Mice and Its Associated Metabolic Alterations
Source: Nutrients. 2026 Aug 17;18(16):2683. doi: 10.3390/nu18162683 (PMC13516763; doi:10.3390/nu18162683)
Supplement: Supplementary file 1 [file nutrients-18-02683-s001.zip › nutrients-4463937-supplementary.pdf]

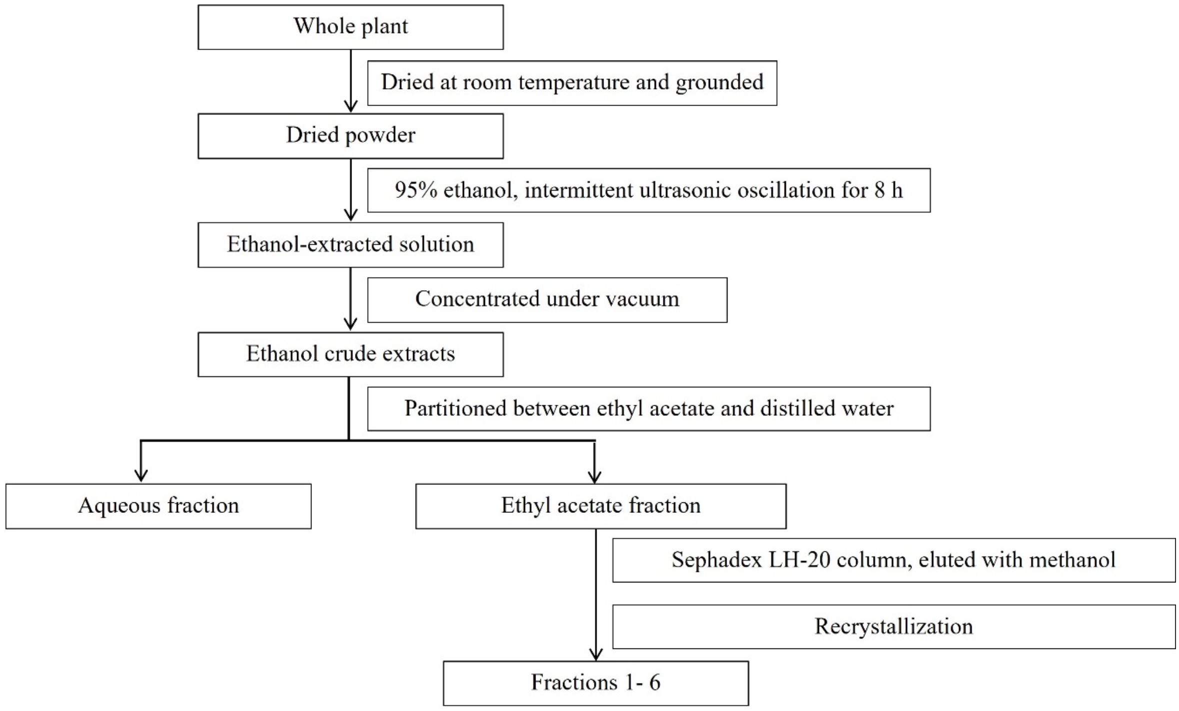

Supplementary Figure S1. Flowchart illustrating the extraction and fractionation of *Erodium stephanianum*.

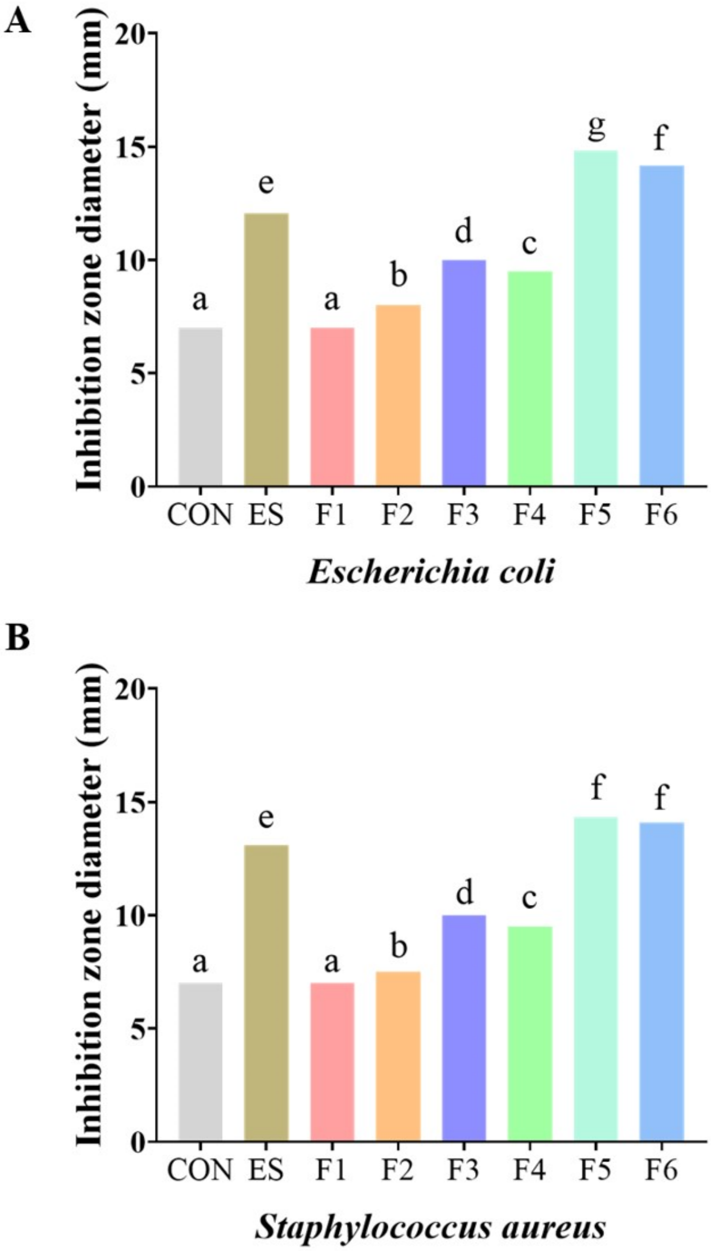

Supplementary Figure S2. Antibacterial activities of the whole extract of *Erodium stephanianum* (ES) and its fractions (F1-F6) against *Escherichia coli* (A) and *Staphylococcus aureus* (B). The inhibition zone diameters (mm) were determined using the agar diffusion method. The values included the diameter of the Oxford cup (7 mm); thus, the negative control (CON) showed the baseline cup diameter without any inhibitory effect. Data are presented as mean values (n = 3). The SE values are negligible and therefore not displayed. Statistical significance was determined by one-way ANOVA followed by Tukey's post hoc test. Different letters indicate statistically significant differences among groups ($P < 0.05$).

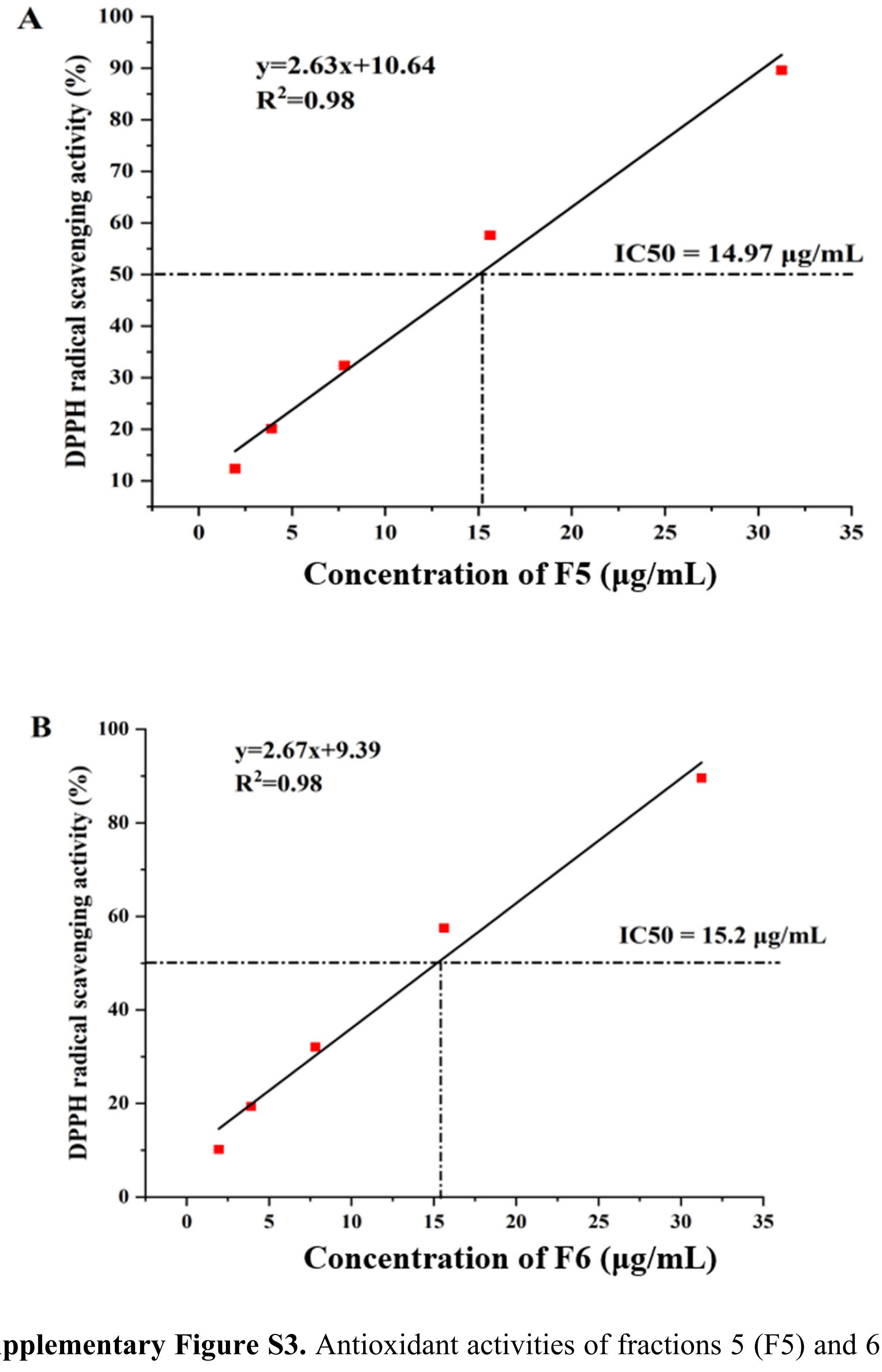

Supplementary Figure S3. Antioxidant activities of fractions 5 (F5) and 6 (F6) from the *Erodium stephanianum* extract.

Supplementary Table S1. Differential metabolic pathways between the CON and Model groups.

Pathway	Total	Hits.total	Hits.sig	Expected	P(Fisher)	P(Gamma)	Enrichment factor
Galactose metabolism	27	2	2	0.41	＜0.01	＜0.01	4.88
Cysteine and methionine metabolism	33	3	2	0.50	0.02	＜0.01	4.00
Fructose and mannose metabolism	18	1	1	0.27	0.08	1.00	3.66
Inositol phosphate metabolism	21	1	1	0.32	0.08	1.00	3.14
Folate biosynthesis	26	3	1	0.39	0.22	1.00	2.54
Pentose and glucuronate interconversions	18	4	1	0.27	0.28	1.00	3.66
Pentose phosphate pathway	23	5	1	0.35	0.34	1.00	2.87
Purine metabolism	71	6	1	1.08	0.40	1.00	0.93
Arginine and proline metabolism	35	6	1	0.53	0.40	1.00	1.88
Nicotinate and nicotinamide metabolism	15	7	1	0.23	0.45	1.00	4.40
Lysine degradation	21	8	1	0.32	0.49	1.00	3.14
Tryptophan metabolism	41	12	1	0.62	0.64	1.00	1.61

Supplementary Table S2. Differential metabolic pathways between the Model and ES-H groups.

Pathway	Total	Hits.total	Hits.sig	Expected	P(Fisher)	P(Gamma)	Enrichment factor
Valine, leucine and isoleucine biosynthesis	8	4	3	0.14	＜0.01	＜0.01	21.02
Glycine, serine and threonine metabolism	31	2	2	0.55	＜0.01	＜0.01	3.62
Pantothenate and CoA biosynthesis	18	4	2	0.32	0.04	＜0.01	6.23
Valine, leucine and isoleucine degradation	35	10	3	0.62	0.06	＜0.01	4.80
Histidine metabolism	16	5	2	0.29	0.07	＜0.01	7.01
Fructose and mannose metabolism	18	1	1	0.32	0.09	1.00	3.11
Inositol phosphate metabolism	21	1	1	0.37	0.09	1.00	2.67
Terpenoid backbone biosynthesis	14	6	2	0.25	0.10	＜0.01	8.01
Galactose metabolism	27	2	1	0.48	0.18	1.00	2.08
D-Amino acid metabolism	15	2	1	0.27	0.18	1.00	3.74
Glycerophospholipid metabolism	13	2	1	0.23	0.18	1.00	4.31
One carbon pool by folate	26	2	1	0.46	0.18	1.00	2.16
Arginine biosynthesis	14	3	1	0.25	0.26	1.00	4.00
Cysteine and methionine metabolism	33	3	1	0.59	0.26	1.00	1.70
beta-Alanine metabolism	21	3	1	0.37	0.26	1.00	2.67
Caffeine metabolism	12	4	1	0.21	0.33	1.00	4.67
Tyrosine metabolism	42	14	2	0.75	0.39	0.02	2.67
Purine metabolism	71	6	1	1.27	0.45	1.00	0.79
Alanine, aspartate and glutamate metabolism	28	6	1	0.50	0.45	1.00	2.00
Arginine and proline metabolism	35	6	1	0.62	0.45	1.00	1.60
Nicotinate and nicotinamide metabolism	15	7	1	0.27	0.50	1.00	3.74

Supplementary Table S3. Differential metabolic pathways between the Model and ES-L groups.

Pathway	Total	Hits.total	Hits.sig	Expected	P(Fisher)	P(Gamma)	Enrichment factor
D-Amino acid metabolism	15	2	2	0.35	0.01	＜0.01	5.75
Arginine biosynthesis	14	3	2	0.32	0.04	＜0.01	6.16
Glutathione metabolism	19	4	2	0.44	0.07	＜0.01	4.54
Pantothenate and CoA biosynthesis	18	4	2	0.42	0.07	＜0.01	4.79
Histidine metabolism	16	5	2	0.37	0.11	＜0.01	5.39
Purine metabolism	71	6	2	1.65	0.16	＜0.01	1.21
Alanine, aspartate and glutamate metabolism	28	6	2	0.65	0.16	＜0.01	3.08
Tyrosine metabolism	42	14	2	0.97	0.53	0.04	2.05
Pentose phosphate pathway	23	5	1	0.53	0.48	1.00	1.87
Ubiquinone and other terpenoid-quinone biosynthesis	7	4	1	0.16	0.41	1.00	6.16
Glycine, serine and threonine metabolism	31	2	1	0.72	0.23	1.00	1.39
Valine, leucine and isoleucine degradation	35	10	1	0.81	0.74	1.00	1.23
Valine, leucine and isoleucine biosynthesis	8	4	1	0.19	0.41	1.00	5.39
Arginine and proline metabolism	35	6	1	0.81	0.55	1.00	1.23
beta-Alanine metabolism	21	3	1	0.49	0.32	1.00	2.05
Inositol phosphate metabolism	21	1	1	0.49	0.12	1.00	2.05
Arachidonic acid metabolism	42	15	1	0.97	0.87	1.00	1.23
Glyoxylate and dicarboxylate metabolism	31	4	1	0.72	0.41	1.00	1.39
Butanoate metabolism	15	4	1	0.35	0.41	1.00	2.87
One carbon pool by folate	26	2	1	0.60	0.23	1.00	1.66
Nicotinate and nicotinamide metabolism	15	7	1	0.35	0.60	1.00	2.87
Porphyrin metabolism	29	5	1	0.67	0.48	1.00	1.49

Nitrogen metabolism	6	1	1	0.14	0.12	1.00	7.19
Metabolism of xenobiotics by cytochrome P450	64	9	1	1.48	0.70	1.00	0.67
Drug metabolism - cytochrome P450	21	5	1	0.49	0.48	1.00	2.05

Supplementary Table S4. Differential metabolic pathways between the Model and Ellagic acid groups.

Pathway	Total	Hits.total	Hits.sig	Expected	P(Fisher)	P(Gamma)	Enrichment factor
Histidine metabolism	16	5	3	0.26	＜0.01	＜0.01	12.36
D-Amino acid metabolism	15	2	2	0.24	＜0.01	＜0.01	8.79
Arginine biosynthesis	14	3	2	0.22	0.02	＜0.01	9.42
Pantothenate and CoA biosynthesis	18	4	2	0.29	0.04	＜0.01	7.33
Purine metabolism	71	6	2	1.14	0.08	＜0.01	1.85
Alanine, aspartate and glutamate metabolism	28	6	2	0.45	0.08	＜0.01	4.71
Inositol phosphate metabolism	21	1	1	0.34	0.08	1.00	3.14
Nitrogen metabolism	6	1	1	0.10	0.08	1.00	10.99
Glycine, serine and threonine metabolism	31	2	1	0.50	0.16	1.00	2.13
One carbon pool by folate	26	2	1	0.42	0.16	1.00	2.54
Cysteine and methionine metabolism	33	3	1	0.53	0.23	1.00	2.13
beta-Alanine metabolism	21	3	1	0.34	0.23	1.00	3.14
Ubiquinone and other terpenoid-quinone biosynthesis	7	4	1	0.11	0.30	1.00	9.42
Valine, leucine and isoleucine biosynthesis	8	4	1	0.13	0.30	1.00	8.24
Glutathione metabolism	19	4	1	0.31	0.30	1.00	3.47
Glyoxylate and dicarboxylate metabolism	31	4	1	0.50	0.30	1.00	2.13
Butanoate metabolism	15	4	1	0.24	0.30	1.00	4.40
Pentose phosphate pathway	23	5	1	0.37	0.36	1.00	2.87
Porphyrin metabolism	29	5	1	0.47	0.36	1.00	2.27
Arginine and proline metabolism	35	6	1	0.56	0.42	1.00	1.88
Nicotinate and nicotinamide metabolism	15	7	1	0.24	0.47	1.00	4.40
Metabolism of xenobiotics by cytochrome P450	64	9	1	1.03	0.56	1.00	1.03

Valine, leucine and isoleucine degradation	35	10	1	0.56	0.59	1.00	1.88
Tyrosine metabolism	42	14	1	0.67	0.72	1.00	1.57

Supplementary Table S5. The differential metabolites between the CON and Model groups in the serum.

Metabolite	Fold change	FDR adjusted P
Methylmalondialdehyde	1.81	4.28E-02
PC(18:3(9,11,15)-OH(13)/P-16:0)	0.35	4.28E-02
PC(22:6/14:0)	0.20	4.28E-02

Supplementary Table S6. The differential metabolites between the ES-L and Model groups in the serum.

Metabolite	Fold change	FDR adjusted P
Sitosterol-beta	0.79	2.39E-02
Methionyl-Leucine	0.69	3.91E-02
Spermic acid 1	2.24	3.57E-02
Lablaboside F	0.75	2.39E-02
Phylloquinone	0.81	2.39E-02
PC(O-16:0/20:3)	6.15	2.39E-02
PE-NMe2(24:1/22:1)	0.78	2.39E-02
alpha-Amyrin cerotate	0.77	2.39E-02
(+)-cis-abscisic aldehyde	0.64	3.01E-02
Trihexosylceramide (d18:1/26:1)	0.75	2.39E-02
TG(14:0/O-18:0/18:1)	0.83	2.39E-02
TG(14:0/18:2/20:4)	0.79	3.78E-02
TG(18:2/20:3n6/22:5)	0.82	2.39E-02
TG(18:0/22:2/22:6)	0.75	2.39E-02
TG(15:0/O-18:0/16:1)	0.78	2.39E-02
TG(14:1/20:4/O-18:1)	0.80	3.47E-02
TG(17:0/18:1/18:1)	0.77	2.39E-02
TG(15:0/18:2/20:1)	0.78	3.78E-02
TG(15:0/22:5/O-18:1)	0.76	3.78E-02
TG(16:1/22:6/22:6)	0.75	4.99E-02

Supplementary Table S7. The differential metabolites between the Ellagic acid and Model groups in the serum.

Metabolite	Fold change	FDR adjusted P
Sitosterol-beta	0.71	4.02E-04
5-phosphonooxy-L-lysine	2.11	1.36E-02
Histidylglutamic acid	0.56	2.08E-03
gamma-Glutamyl-5-hydroxytryptophan	0.39	4.64E-02
Putrescine	1.97	2.89E-02
Methionyl-Leucine	0.63	1.23E-02
N1,N12-Diacetylspermine	0.45	3.71E-02
Tetrahydrodipicolinate	1.44	4.36E-02
Spermic acid 1	2.08	5.47E-03
2-Oxothiazolidine-4-carboxylic acid	0.79	6.93E-03
N(1),N(12)-diacetylsperminium(2+)	0.45	3.71E-02
Lablaboside F	0.66	1.15E-02
DG(TXB2/0:0/10:0)	0.54	2.31E-02
DG(i-12:0/0:0/22:6-2OH(10S,17))	0.54	2.17E-02
Semidehydroascorbic acid	0.54	2.38E-03
Aluminum palmitate	1.44	3.56E-02
Phlorin	0.59	2.74E-02
Phylloquinone	0.73	4.02E-04
Dopaminechrome	1.95	4.73E-02
2,3,5-Trichloromaleylacetate	0.79	3.00E-02
PC(O-16:0/20:3)	8.53	5.07E-03
PC(18:1/18:2)	1.78	4.96E-02

PC(18:1-O(12,13)/17:0)	0.60	1.60E-02
PE-NMe2(22:1/15:0)	23.25	2.53E-02
PC(20:3(6,8,11)-OH(5)/17:0)	0.29	5.35E-03
PC(P-18:1/22:5)	4.13	9.76E-03
PC(TXB2/P-16:0)	0.65	9.39E-03
PC(20:5(6E,8Z,11Z,14Z,17Z)-OH(5)/DiMe(9,3))	5.49	1.25E-02
PE-NMe(24:1/18:4)	3.60	3.71E-02
PE-NMe2(24:1/22:0)	0.71	4.02E-04
PC(24:0/24:0)	0.72	3.28E-02
Lutein diacetate	2.22	1.08E-02
alpha-Amyrin cerotate	0.70	4.02E-04
(+)-cis-abscisic aldehyde	0.47	7.10E-04
Dihydrobiopterin	0.45	1.84E-02
2-O-Methyl-L-fucose	0.70	4.23E-02
Benzyl sulfate	0.59	6.40E-03
p-Coumaric acid sulfate	0.78	5.18E-03
Homovanillyl alcohol sulfate	4.30	2.28E-02
Trihexosylceramide (d18:1/26:1)	0.67	4.77E-03
Galactosylceramide (d18:1/22:0)	0.69	4.78E-02
Ganglioside GA2 (d18:1/9-18:1)	0.76	1.46E-03
TG(14:0/O-18:0/18:1)	0.76	4.15E-04
TG(14:0/18:2/20:4)	0.70	5.87E-03
TG(18:2/20:3n6/22:5)	0.75	4.02E-04

TG(18:0/22:2/22:6)	0.66	1.46E-03
TG(15:0/O-18:0/16:1)	0.72	4.02E-04
TG(15:0/18:2/O-18:0)	0.77	2.74E-02
TG(15:0/O-18:0/18:2)	0.76	1.73E-02
TG(14:1/20:4/O-18:0)	0.73	7.11E-04
TG(14:1/22:5/O-18:0)	0.75	6.93E-03
TG(17:0/18:1/18:1)	0.70	4.02E-04
TG(15:0/18:2/20:0)	0.70	4.02E-04
TG(16:0/16:0/22:5)	0.69	1.57E-02
TG(16:0/18:4/20:1)	0.69	9.20E-03
TG(15:0/22:5/O-18:0)	0.69	5.47E-03
TG(16:0/22:5/20:2n6)	0.77	2.31E-02
TG(16:1/22:6/22:6)	0.70	2.73E-02
TG(18:3/20:4/22:6)	0.68	1.73E-02

Supplementary Table S8. The differential metabolites between the ES-H and Ellagic acid groups in the serum.

Metabolite	Fold change	FDR adjusted P
5-phosphonooxy-L-lysine	1.61	3.98E-02
TG(18:2/20:3n6/22:5)	0.83	3.98E-02
Ganglioside GA2 (d18:1/18:1)	0.84	3.98E-02
PC(20:5-OH/DiMe)	5.19	3.98E-02
